# Supplementary material for: Social conditions and mental health during COVID-19 lockdown among people who do not identify with the man/woman binomial in Spain
Source: PLoS One. 2021 Aug 20;16(8):e0256261. doi: 10.1371/journal.pone.0256261 (PMC8378716; doi:10.1371/journal.pone.0256261)
Supplement: S1 Table — (DOCX) [file pone.0256261.s001.docx]

**S1 Table**. Mental health, sociodemographic characteristics and social and health-related factors among people who do not identify within the man/woman binomial during COVID-19 lockdown in Spain.

|  | **Non-binary/**  **I do not identify**  **(n=72)** | **Matched Men/Women**  **(n=288)** | **P-value^3^** |
| --- | --- | --- | --- |
| **GAD-7^1^** |  |  |  |
| Normal/Mild | 42 (58.3%) | 210 (72.9%) | 0.016 |
| Moderate/Severe | 30 (41.7%) | 78 (27.1%) |  |
| **PHQ-9^2^** |  |  |  |
| None-minimal/Mild | 50 (69.4%) | 205 (71.2%) | 0.772 |
| Moderate/Moderately severe/Severe | 22 (30.6%) | 83 (28.8%) |  |
| **Age** |  |  |  |
| 18-35 years | 26 (36.1%) | 103 (35.8%) | 0.956 |
| >35 years | 46 (63.9%) | 185 (64.2%) |  |
| **Educational level** |  |  |  |
| Primary/Secondary | 23 (32.4%) | 92 (31.9%) | 1.000 |
| University | 48 (67.6%) | 196 (68.1%) |  |
| **Country of Birth** |  |  |  |
| Spain | 64 (88.9%) | 259 (89.9%) | 0.795 |
| Other countries | 8 (11.1%) | 29 (10.1%) |  |
| **Employment status before lockdown** |  |  |  |
| Working | 52 (72.2%) | 208 (72.2%) | 1.000 |
| Not working | 20 (27.8%) | 80 (27.8%) |  |
| **Essential work** |  |  |  |
| No | 58 (80.6%) | 213 (74.0%) | 0.246 |
| Yes | 14 (19.4%) | 75 (26.0%) |  |
| **Employment condition** |  |  |  |
| No change/Improved | 35 (48.6%) | 156 (54.2%) | 0.398 |
| Worsened | 37 (51.4%) | 132 (45.8%) |  |
| **Living conditions** |  |  |  |
| Alone | 15 (20.8%) | 54 (18.8%) | 0.688 |
| Not alone | 57 (79.2%) | 234 (81.3%) |  |
| **Adequate housing conditions** |  |  |  |
| No | 10 (13.9%) | 34 (11.8%) | 0.629 |
| Yes | 62 (86.1%) | 254 (88.2%) |  |
| **Concern relationships with people live with** |  |  |  |
| No | 51 (71.8%) | 225 (78.1%) | 0.260 |
| Yes | 20 (28.2%) | 63 (21.9%) |  |
| **Violence at home** |  |  |  |
| No | 65 (91.5%) | 281 (97.6%) | 0.015 |
| Yes | 6 (8.5%) | 7 (2.4%) |  |
| **Self-rated health** |  |  |  |
| Good/Very good/Excellent | 63 (87.5%) | 260 (90.3%) | 0.488 |
| Regular /Poor | 9 (12.5%) | 28 (9.7%) |  |
| **COVID-19 diagnostic or symptoms** |  |  |  |
| No | 63 (87.5%) | 229 (79.5%) | 0.122 |
| Yes | 9 (12.5%) | 59 (20.5%) |  |
| **Dead of loved ones** |  |  |  |
| No | 58 (80.6%) | 249 (86.5%) | 0.206 |
| Yes | 14 (19.4%) | 39 (13.5%) |  |
| **Support from neighbours** |  |  |  |
| No | 16 (22.9%) | 40 (15.2%) | 0.125 |
| Yes | 54 (77.1%) | 224 (84.8%) |  |
| Fear of COVID-19 infection |  |  |  |
| No | 32 (44.4%) | 120 (41.7%) | 0670 |
| Yes | 40 (55.6%) | 168 (58.3%) |  |
| **COVID-19 is a problem for your economy** |  |  |  |
| No | 13 (18.1%) | 61 (21.2%) | 0.557 |
| Yes | 59 (81.9%) | 227 (78.8%) |  |
| **Tobacco consumption** |  |  |  |
| No use/Same use | 62 (86.1%) | 241 (83.7%) | 0.248 |
| Increased use | 8 (11.1%) | 24 (8.3%) |  |
| Decreased use | 2 (2.8%) | 23 (8.0%) |  |
| **Alcohol consumption** |  |  |  |
| No use/Same use | 43 (59.7%) | 208 (72.2%) | 0.082 |
| Increased use | 14 (19.4%) | 32 (11.1%) |  |
| Decreased use | 15 (20.8%) | 48 (16.7%) |  |
| **Practice physical activity** |  |  |  |
| No practice/Same practice | 33 (45.8%) | 80 (27.8%) | 0.011 |
| Increased practice | 10 (13.9%) | 63 (21.9%) |  |
| Decreased practice | 29 (40.3%) | 145 (50.3%) |  |

^1^ GAD 7: Generalised Anxiety Disorder 7-item scale ^2^ PHQ-9: Patient Health Questionnaire

^3^Chi-Square test
