# Supplementary material for: Social conditions and mental health during COVID-19 lockdown among people who do not identify with the man/woman binomial in Spain
Source: PLoS One. 2021 Aug 20;16(8):e0256261. doi: 10.1371/journal.pone.0256261 (PMC8378716; doi:10.1371/journal.pone.0256261)
Supplement: S2 Table — (DOCX) [file pone.0256261.s002.docx]

**S2 Table**. Mental health, sociodemographic characteristics and social and health-related factors among people who do not identify within the man/woman binomial and men and women during COVID-19 lockdown in Spain.

|  | **Non-binary/**  **I do not identify**  **(n=72)** | **Matched Men**  **(n=144)** | **Matched Women**  **(n=144)** |  | **P-value**  **(Non-Binary / Men)^3^** | **P-value**  **(Non-Binary / Women)^3^** |
| --- | --- | --- | --- | --- | --- | --- |
| **GAD-7^1^** |  |  |  |  |  |  |
| Normal/Mild | 42 (58.3%) | 121 (84.0%) | 89 (61.8%) |  | <0.001 | 0.622 |
| Moderate/Severe | 30 (41.7%) | 23 (16.0%) | 55 (38.2%) |  |  |  |
| **PHQ-9^2^** |  |  |  |  |  |  |
| None-minimal/Mild | 50 (69.4%) | 117 (81.3%) | 88 (61.1%) |  | 0.051 | 0.229 |
| Moderate/Moderately severe/Severe | 22 (30.6%) | 27 (18.8%) | 56 (38.9%) |  |  |  |
| **Age** |  |  |  |  |  |  |
| 18-35 years | 26 (36.1%) | 51 (35.4%) | 52 (36.1%) |  | 0.920 | 1.000 |
| >35 years | 46 (63.9%) | 93 (64.6%) | 92 (63.9%) |  |  |  |
| **Educational level** |  |  |  |  |  |  |
| Primary/Secondary | 23 (32.4%) | 46 (31.9%) | 46 (31.9%) |  | 1.000 | 1.000 |
| University | 48 (67.6%) | 98 (68.1%) | 98 (68.1%) |  |  |  |
| **Country of Birth** |  |  |  |  |  |  |
| Spain | 64 (88.9%) | 132 (91.7%) | 127 (88.2%) |  | 0.507 | 0.880 |
| Other countries | 8 (11.1%) | 12 (8.3%) | 17 (11.8%) |  |  |  |
| **Employment status before lockdown** |  |  |  |  |  |  |
| Working | 52 (72.2%) | 109 (75.7%) | 99 (68.8%) |  | 0.581 | 0.600 |
| Not working | 20 (27.8%) | 35 (24.3%) | 45 (31.3%) |  |  |  |
| **Essential work** |  |  |  |  |  |  |
| No | 58 (80.6%) | 112 (77.8%) | 101 (70.1%) |  | 0.638 | 0.102 |
| Yes | 14 (19.4%) | 32 (22.2%) | 43 (29.9%) |  |  |  |
| **Employment condition** |  |  |  |  |  |  |
| No change/Improved | 35 (48.6%) | 75 (52.1%) | 81 (56.3%) |  | 0.630 | 0.289 |
| Worsened | 37 (51.4%) | 69 (47.9%) | 63 (43.8%) |  |  |  |
| **Living conditions** |  |  |  |  |  |  |
| Alone | 15 (20.8%) | 32 (22.2%) | 22 (15.3%) |  | 0816 | 0.307 |
| Not alone | 57 (79.2%) | 112 (77.8%) | 122 (84.7%) |  |  |  |
| **Adequate housing conditions** |  |  |  |  |  |  |
| No | 10 (13.9%) | 20 (13.9%) | 14 (9.7%) |  | 1.000 | 0.351 |
| Yes | 62 (86.1%) | 124 (86.1%) | 130 (90.3%) |  |  |  |
| **Concern relationships with people live with** |  |  |  |  |  |  |
| No | 51 (71.8%) | 119 (82.6%) | 106 (73.6%) |  | 0.067 | 0.782 |
| Yes | 20 (28.2%) | 25 (17.4%) | 38 (26.4%) |  |  |  |
| **Violence at home** |  |  |  |  |  |  |
| No | 65 (91.5%) | 142 (98.6%) | 139 (96.5%) |  | 0.010 | 0.119 |
| Yes | 6 (8.5%) | 2 (1.4%) | 5 (3.5%) |  |  |  |
| **Self-rated health** |  |  |  |  |  |  |
| Good/Very good/Excellent | 63 (87.5%) | 132 (91.7%) | 128 (88.9%) |  | 0.330 | 0.764 |
| Regular /Poor | 9 (12.5%) | 12 (8.3%) | 16 (11.1%) |  |  |  |
| **COVID-19 diagnostic or symptoms** |  |  |  |  |  |  |
| No | 63 (87.5%) | 112 (77.8%) | 117 (81.3%) |  | 0.086 | 0.245 |
| Yes | 9 (12.5%) | 32 (22.2%) | 27 (18.8%) |  |  |  |
| **Dead of loved ones** |  |  |  |  |  |  |
| No | 58 (80.6%) | 126 (87.5%) | 123 (85.4%) |  | 0.176 | 0.361 |
| Yes | 14 (19.4%) | 18 (12.5%) | 21 (14.6%) |  |  |  |
| **Support from neighbours** |  |  |  |  |  |  |
| No | 16 (22.9%) | 18 (13.7%) | 22 (16.5%) |  | 0.100 | 0.273 |
| Yes | 54 (77.1%) | 113 (86.3%) | 111 (83.5%) |  |  |  |
| Fear of COVID-19 infection |  |  |  |  |  |  |
| No | 32 (44.4%) | 65 (45.1%) | 55 (38.2%) |  | 0.923 | 0.377 |
| Yes | 40 (55.6%) | 79 (54.9%) | 89 (61.8%) |  |  |  |
| **COVID-19 is a problem for your economy** |  |  |  |  |  |  |
| No | 13 (18.1%) | 26 (18.1%) | 35 (24.3%) |  | 1.000 | 0.298 |
| Yes | 59 (81.9%) | 118 (81.9%) | 109 (75.7%) |  |  |  |
| **Tobacco consumption** |  |  |  |  |  |  |
| No use/Same use | 62 (86.1%) | 122 (84.7%) | 119 (82.6%) |  | 0.077 | 0.547 |
| Increased use | 8 (11.1%) | 8 (5.6%) | 16 (11.1%) |  |  |  |
| Decreased use | 2 (2.8%) | 14 (9.7%) | 9 (6.3%) |  |  |  |
| **Alcohol consumption** |  |  |  |  |  |  |
| No use/Same use | 43 (59.7%) | 98 (68.1%) | 110 (76.4%) |  | 0.132 | 0.037 |
| Increased use | 14 (19.4%) | 14 (9.7%) | 18 (12.5%) |  |  |  |
| Decreased use | 15 (20.8%) | 32 (22.2%) | 16 (11.1%) |  |  |  |
| **Practice physical activity** |  |  |  |  |  |  |
| No practice/Same practice | 33 (45.8%) | 41 (28.5%) | 39 (27.1%) |  | 0.037 | 0.009 |
| Increased practice | 10 (13.9%) | 23 (16.0%) | 40 (27.8%) |  |  |  |
| Decreased practice | 29 (40.3%) | 80 (55.6%) | 65 (45.1%) |  |  |  |

^1^ GAD 7: Generalised Anxiety Disorder 7-item scale ^2^ PHQ-9: Patient Health Questionnaire

^3^Chi-Square test
