# Supplementary material for: Social conditions and mental health during COVID-19 lockdown among people who do not identify with the man/woman binomial in Spain
Source: PLoS One. 2021 Aug 20;16(8):e0256261. doi: 10.1371/journal.pone.0256261 (PMC8378716; doi:10.1371/journal.pone.0256261)
Supplement: S3 Table — (DOCX) [file pone.0256261.s003.docx]

**S3 Table.** Associations between sociodemographic characteristics, gender identity, social and health-related factors, and mental health during COVID-19 lockdown in Spain

|  | **GAD7^1^** | | **PHQ-9^2^** | |
| --- | --- | --- | --- | --- |
|  | **aOR^3^ (95%CI)** | **P-value^4^** | **aOR^3^ (95%CI)** | **P-value** |
| **Age** |  |  |  |  |
| >35 years | 1.00 |  | 1.00 |  |
| 18-35 age | 1.61 (0.96-2.70) | 0.069 | 2.57 (1.53-4.34) | 0.000 |
| **Gender** |  |  |  |  |
| Men/Women | 1.00 |  | 1.00 |  |
| Non-binary/Not identify | 1.89 (1.05-3.39) | 0.032 | 0.93 (0.50-1.74) | 0.821 |
| **Educational level** |  |  |  |  |
| Primary/Secondary | 1.00 |  | 1.00 |  |
| University | 0.92 (0.52-1.62) | 0.763 | 0.94 (0.53-1.67) | 0.829 |
| **Employment status before lockdown** |  |  |  |  |
| Working | 1.00 |  | 1.00 |  |
| Not working | 1.00 (0.55-1.83) | 0.999 | 1.98 (1.09-3.58) | 0.024 |
| **Employment condition** |  |  |  |  |
| No change/Improved | 1.00 |  | 1.00 |  |
| Worsened | 2.25 (1.37-3.70) | 0.001 | 1.83 (1.11-3.03) | 0.019 |
| **Self-rated health** |  |  |  |  |
| Good/Very good/Excellent | 1.00 |  | 1.00 |  |
| Regular /Poor | 6.46 (2.95-14.15) | 0.000 | 7.69 (3.49-16.96) | 0.000 |
| **Fear of COVID-19 infection** |  |  |  |  |
| No | 1.00 |  | 1.00 |  |
| Yes | 2.02 (1.20-3.39) | 0.008 | 1.41 (0.84-2.37) | 0.190 |
| **Alcohol consumption** |  |  |  |  |
| No use/Same use | 1.00 |  | 1.00 |  |
| Increased use | 1.30 (0.61-2.78) |  | 2.35 (1.11-4.96) |  |
| Decreased use | 1.19 (0.62-2.29) | 0.730 | 1.32 (0.69-2.53) | 0.074 |

Models were adjusted for age, education level, pre-lockdown employment status, employment condition as consequence of lockdown, self-rated health, fear of being infected with COVID-19 and changes in alcohol consumption during lockdown.

^1^ GAD 7: Generalised Anxiety Disorder 7-item scale

^2^ PHQ-9: Patient Health Questionnaire

^3^aOR: adjusted ordinal odds ratio.

^4^P-value: Statistical significance derived from using Wald test
