# Supplementary material for: Social conditions and mental health during COVID-19 lockdown among people who do not identify with the man/woman binomial in Spain
Source: PLoS One. 2021 Aug 20;16(8):e0256261. doi: 10.1371/journal.pone.0256261 (PMC8378716; doi:10.1371/journal.pone.0256261)
Supplement: S4 Table — (DOCX) [file pone.0256261.s004.docx]

**S4 Table**. GAD-7^1^ Items.

|  | **Non-binary/**  **not identify**  **(n=72)** | **Matched Men/Women**  **(n=288)** | **P-value^2^** |
| --- | --- | --- | --- |
| **Feeling nervous, anxious or on edge** |  |  |  |
| Not at all | 18 (25.0%) | 81 (28.1%) | 0.862 |
| Several days | 30 (41.7%) | 123 (42.7%) |  |
| More than half the days | 16 (22.2%) | 52 (18.1%) |  |
| Nearly every day | 8 (11.1%) | 32 (11.1%) |  |
| **Not being able to stop or control worrying** |  |  |  |
| Not at all | 20 (27.8%) | 89 (30.9%) | 0.915 |
| Several days | 24 (33.3%) | 99 (34.4%) |  |
| More than half the days | 16 (22.2%) | 59 (20.5%) |  |
| Nearly every day | 12 (16.7%) | 41 (14.2%) |  |
| **Worrying too much about different things** |  |  |  |
| Not at all | 20 (27.8%) | 93 (32.3%) | 0.656 |
| Several days | 25 (34.7%) | 109 (37.8%) |  |
| More than half the days | 17 (23.6%) | 53 (18.4%) |  |
| Nearly every day | 10 (13.9%) | 33 (11.5%) |  |
| **Having trouble relaxing** |  |  |  |
| Not at all | 20 (27.8%) | 89 (30.9%) | 0.653 |
| Several days | 25 (34.7%) | 111 (38.5%) |  |
| More than half the days | 14 (19.4%) | 51 (17.7%) |  |
| Nearly every day | 13 (18.1%) | 37 (12.8%) |  |
| **Being so restless that it is hard to sit still** |  |  |  |
| Not at all | 31 (43.1%) | 152 (52.8%) | 0.015 |
| Several days | 19 (26.4%) | 91 (31.6%) |  |
| More than half the days | 16 (22.2%) | 26 (9.0%) |  |
| Nearly every day | 6 (8.3%) | 19 (6.6%) |  |
| **Becoming easily annoyed or irritable** |  |  |  |
| Not at all | 19 (26.4%) | 110 (38.2%) | 0.234 |
| Several days | 30 (41.7%) | 101 (35.1%) |  |
| More than half the days | 14 (19.4%) | 54 (18.8%) |  |
| Nearly every day | 9 (12.5%) | 23 (8.0%) |  |
| **Feeling afraid as if something awful might happen** |  |  |  |
| Not at all | 34 (47.2%) | 156 (54.2%) | 0.679 |
| Several days | 23 (31.9%) | 84 (29.2%) |  |
| More than half the days | 8 (11.1%) | 29 (10.1%) |  |
| Nearly every day | 7 (9.7%) | 19 (6.6%) |  |

^1^ GAD 7: Generalised Anxiety Disorder 7-item scale

^2^Chi-Square test
