# Supplementary material for: Social conditions and mental health during COVID-19 lockdown among people who do not identify with the man/woman binomial in Spain
Source: PLoS One. 2021 Aug 20;16(8):e0256261. doi: 10.1371/journal.pone.0256261 (PMC8378716; doi:10.1371/journal.pone.0256261)
Supplement: S5 Table — (DOCX) [file pone.0256261.s005.docx]

**S5 Table**. PHQ-9^1^ Items.

|  | **Non-binary/**  **not identify**  **(n=72)** | **Matched Men/Women**  **(n=288)** | **P-value^2^** |
| --- | --- | --- | --- |
| **Little interest or pleasure in doing things** |  |  |  |
| Not at all | 25 (34.7%) | 85 (29.5%) | 0.789 |
| Several days | 27 (37.5%) | 123 (42.7%) |  |
| More than half the days | 11 (15.3%) | 48 (16.7%) |  |
| Nearly every day | 9 (12.5%) | 32 (11.1%) |  |
| **Feeling down, depressed, or hopeless** |  |  |  |
| Not at all | 26 (36.1%) | 123 (42.7%) | 0.132 |
| Several days | 30 (41.7%) | 102 (35.4%) |  |
| More than half the days | 7 (9.7%) | 45 (15.6%) |  |
| Nearly every day | 9 (12.5%) | 18 (6.3%) |  |
| **Trouble falling or staying asleep, or sleeping too much** |  |  |  |
| Not at all | 25 (34.7%) | 115 (39.9%) | 0.212 |
| Several days | 16 (22.2%) | 73 (25.3%) |  |
| More than half the days | 13 (18.1%) | 58 (20.1%) |  |
| Nearly every day | 18 (25.0%) | 42 (14.6%) |  |
| **Feeling tired or having little energy** |  |  |  |
| Not at all | 21 (29.2%) | 87 (30.2%) | 0.830 |
| Several days | 28 (38.9%) | 116 (40.3%) |  |
| More than half the days | 13 (18.1%) | 56 (19.4%) |  |
| Nearly every day | 10 (13.9%) | 29 (10.1%) |  |
| **Poor appetite or overeating** |  |  |  |
| Not at all | 30 (41.7%) | 110 (38.2%) | 0.648 |
| Several days | 19 (26.4%) | 88 (30.6%) |  |
| More than half the days | 11 (15.3%) | 54 (18.8%) |  |
| Nearly every day | 12 (16.7%) | 36 (12.5%) |  |
| **Feeling bad about yourself - or that you are a failure or have let yourself or your family down** |  |  |  |
| Not at all | 47 (65.3%) | 182 (63.2%) | 0.693 |
| Several days | 16 (22.2%) | 64 (22.2%) |  |
| More than half the days | 3 (4.2%) | 23 (8.0%) |  |
| Nearly every day | 6 (8.3%) | 19 (6.6%) |  |
| **Trouble concentrating on things, such as reading the newspaper or watching television** |  |  |  |
| Not at all | 29 (40.3%) | 127 (44.1%) | 0.771 |
| Several days | 25 (34.7%) | 90 (31.3%) |  |
| More than half the days | 10 (13.9%) | 47 (16.3%) |  |
| Nearly every day | 8 (11.1%) | 24 (8.3%) |  |
| **Moving or speaking so slowly that other people could have noticed. Or the opposite - being so fidgety or restless that you have been moving around a lot more than usual** |  |  |  |
| Not at all | 50 (69.4%) | 226 (78.5%) | 0.428 |
| Several days | 14 (19.4%) | 42 (14.6%) |  |
| More than half the days | 6 (8.3%) | 15 (5.2%) |  |
| Nearly every day | 2 (2.8%) | 5 (1.7%) |  |
| **Thoughts that you would be better off dead or of hurting yourself in some way** |  |  |  |
| Not at all | 65 (90.3%) | 260 (90.3%) | 0.668 |
| Several days | 3 (4.2%) | 17 (5.9%) |  |
| More than half the days | 2 (2.8%) | 8 (2.8%) |  |
| Nearly every day | 2 (2.8%) | 3 (1.0%) |  |

^1^ PHQ-9: Patient Health Questionnaire

^2^Chi-Square test
