# Supplementary material for: Social conditions and mental health during COVID-19 lockdown among people who do not identify with the man/woman binomial in Spain
Source: PLoS One. 2021 Aug 20;16(8):e0256261. doi: 10.1371/journal.pone.0256261 (PMC8378716; doi:10.1371/journal.pone.0256261)
Supplement: S6 Table — (DOCX) [file pone.0256261.s006.docx]

**S6 Table**. GAD-7^1^ Items.

|  | **Non-binary/**  **I do not identify**  **(n=72)** | **Matched Men**  **(n=144)** | **Matched Women**  **(n=144)** |  | **P-value**  **(Non-Binary / Men)^2^** | **P-value**  **(Non-Binary / Women)^2^** |
| --- | --- | --- | --- | --- | --- | --- |
| **Feeling nervous, anxious or on edge** |  |  |  |  |  |  |
| Not at all | 18 (25.0%) | 48 (33.3%) | 33 (22.9%) |  | 0.082 | 0.655 |
| Several days | 30 (41.7%) | 70 (48.6%) | 53 (36.8%) |  |  |  |
| More than half the days | 16 (22.2%) | 19 (13.2%) | 33 (22.9%) |  |  |  |
| Nearly every day | 8 (11.1%) | 7 (4.9%) | 25 (17.4%) |  |  |  |
| **Not being able to stop or control worrying** |  |  |  |  |  |  |
| Not at all | 20 (27.8%) | 52 (36.1%) | 37 (25.7%) |  | 0.155 | 0.852 |
| Several days | 24 (33.3%) | 57 (39.6%) | 42 (29.2%) |  |  |  |
| More than half the days | 16 (22.2%) | 22 (15.3%) | 37 (25.7%) |  |  |  |
| Nearly every day | 12 (16.7%) | 13 (9.0%) | 28 (19.4%) |  |  |  |
| **Worrying too much about different things** |  |  |  |  |  |  |
| Not at all | 20 (27.8%) | 55 (38.2%) | 38 (26.4%) |  | 0.031 | 0.976 |
| Several days | 25 (34.7%) | 61 (42.4%) | 48 (33.3%) |  |  |  |
| More than half the days | 17 (23.6%) | 15 (10.4%) | 38 (26.4%) |  |  |  |
| Nearly every day | 10 (13.9%) | 13 (9.0%) | 20 (13.9%) |  |  |  |
| **Having trouble relaxing** |  |  |  |  |  |  |
| Not at all | 20 (27.8%) | 47 (32.6%) | 42 (29.2%) |  | 0.038 | 0.699 |
| Several days | 25 (34.7%) | 69 (47.9%) | 42 (29.2%) |  |  |  |
| More than half the days | 14 (19.4%) | 14 (9.7%) | 37 (25.7%) |  |  |  |
| Nearly every day | 13 (18.1%) | 14 (9.7%) | 23 (16.0%) |  |  |  |
| **Being so restless that it is hard to sit still** |  |  |  |  |  |  |
| Not at all | 31 (43.1%) | 84 (58.3%) | 68 (47.2%) |  | 0.001 | 0.395 |
| Several days | 19 (26.4%) | 46 (31.9%) | 45 (31.3%) |  |  |  |
| More than half the days | 16 (22.2%) | 7 (4.9%) | 19 (13.2%) |  |  |  |
| Nearly every day | 6 (8.3%) | 7 (4.9%) | 12 (8.3%) |  |  |  |
| **Becoming easily annoyed or irritable** |  |  |  |  |  |  |
| Not at all | 19 (26.4%) | 130 (90.3%) | 45 (31.3%) |  | 0.877 | 0.697 |
| Several days | 30 (41.7%) | 8 (5.6%) | 49 (34%) |  |  |  |
| More than half the days | 14 (19.4%) | 4 (2.8%) | 35 (24.3%) |  |  |  |
| Nearly every day | 9 (12.5%) | 2 (1.4%) | 15 (10.4%) |  |  |  |
| **Feeling afraid as if something awful might happen** |  |  |  |  |  |  |
| Not at all | 34 (47.2%) | 130 (90.3%) | 67 (46.5%) |  | 0.599 | 0.977 |
| Several days | 23 (31.9%) | 9 (6.3%) | 44 (30.6%) |  |  |  |
| More than half the days | 8 (11.1%) | 4 (2.8%) | 19 (13.2%) |  |  |  |
| Nearly every day | 7 (9.7%) | 1 (0.7%) | 14 (9.7%) |  |  |  |

^1^ GAD 7: Generalised Anxiety Disorder 7-item scale

^2^Chi-Square test
