# Supplementary material for: Social conditions and mental health during COVID-19 lockdown among people who do not identify with the man/woman binomial in Spain
Source: PLoS One. 2021 Aug 20;16(8):e0256261. doi: 10.1371/journal.pone.0256261 (PMC8378716; doi:10.1371/journal.pone.0256261)
Supplement: S7 Table — (DOCX) [file pone.0256261.s007.docx]

**S7 Table**. PHQ-9^1^ Items.

|  | **Non-binary/**  **I do not identify**  **(n=72)** | **Matched Men**  **(n=144)** | **Matched Women**  **(n=144)** |  | **P-value**  **(Non-Binary / Men)^2^** | **P-value**  **(Non-Binary / Women)_2_** |
| --- | --- | --- | --- | --- | --- | --- |
| **Little interest or pleasure in doing things** |  |  |  |  |  |  |
| Not at all | 25 (34.7%) | 45 (31.3%) | 40 (27.8%) |  | 0.536 | 0.756 |
| Several days | 27 (37.5%) | 66 (45.8%) | 57 (39.6%) |  |  |  |
| More than half the days | 11 (15.3%) | 22 (15.3%) | 26 (18.1%) |  |  |  |
| Nearly every day | 9 (12.5%) | 11 (7.6%) | 21 (14.6%) |  |  |  |
| **Feeling down, depressed, or hopeless** |  |  |  |  |  |  |
| Not at all | 26 (36.1%) | 71 (49.3%) | 52 (36.1%) |  | 0.023 | 0.155 |
| Several days | 30 (41.7%) | 55 (38.2%) | 47 (32.6%) |  |  |  |
| More than half the days | 7 (9.7%) | 14 (9.7%) | 31 (21.5%) |  |  |  |
| Nearly every day | 9 (12.5%) | 4 (2.8%) | 14 (9.7%) |  |  |  |
| **Trouble falling or staying asleep, or sleeping too much** |  |  |  |  |  |  |
| Not at all | 25 (34.7%) | 68 (47.2%) | 47 (32.6%) |  | 0.024 | 0.707 |
| Several days | 16 (22.2%) | 36 (25.0%) | 37 (25.7%) |  |  |  |
| More than half the days | 13 (18.1%) | 26 (18.1%) | 32 (22.2%) |  |  |  |
| Nearly every day | 18 (25.0%) | 14 (9.7%) | 28 (19.4%) |  |  |  |
| **Feeling tired or having little energy** |  |  |  |  |  |  |
| Not at all | 21 (29.2%) | 53 (36.8%) | 34 (23.6%) |  | 0.041 | 0.540 |
| Several days | 28 (38.9%) | 66 (45.8%) | 50 (34.7%) |  |  |  |
| More than half the days | 13 (18.1%) | 19 (13.2%) | 37 (25.7%) |  |  |  |
| Nearly every day | 10 (13.9%) | 6 (4.2%) | 23 (16.0%) |  |  |  |
| **Poor appetite or overeating** |  |  |  |  |  |  |
| Not at all | 30 (41.7%) | 63 (43.8%) | 47 (32.6%) |  | 0.226 | 0.542 |
| Several days | 19 (26.4%) | 47 (32.6%) | 41 (28.5%) |  |  |  |
| More than half the days | 11 (15.3%) | 23 (16%) | 31 (21.5%) |  |  |  |
| Nearly every day | 12 (16.7%) | 11 (7.6%) | 25 (17.4%) |  |  |  |
| **Feeling bad about yourself - or that you are a failure or have let yourself or your family down** |  |  |  |  |  |  |
| Not at all | 47 (65.3%) | 100 (69.4%) | 82 (56.9%) |  | 0.186 | 0.364 |
| Several days | 16 (22.2%) | 33 (22.9%) | 31 (21.5%) |  |  |  |
| More than half the days | 3 (4.2%) | 8 (5.6%) | 15 (10.4%) |  |  |  |
| Nearly every day | 6 (8.3%) | 3 (2.1%) | 16 (11.1%) |  |  |  |
| **Trouble concentrating on things, such as reading the newspaper or watching television** |  |  |  |  |  |  |
| Not at all | 29 (40.3%) | 66 (45.8%) | 61 (42.4%) |  | 0.505 | 0.687 |
| Several days | 25 (34.7%) | 50 (34.7%) | 40 (27.8%) |  |  |  |
| More than half the days | 10 (13.9%) | 20 (13.9%) | 27 (18.8%) |  |  |  |
| Nearly every day | 8 (11.1%) | 8 (5.6%) | 16 (11.1%) |  |  |  |
| **Moving or speaking so slowly that other people could have noticed. Or the opposite - being so fidgety or restless that you have been moving around a lot more than usual** |  |  |  |  |  |  |
| Not at all | 50 (69.4%) | 115 (79.9%) | 111 (77.1%) |  | 0.336 | 0.688 |
| Several days | 14 (19.4%) | 21 (14.6%) | 21 (14.6%) |  |  |  |
| More than half the days | 6 (8.3%) | 6 (4.2%) | 9 (6.3%) |  |  |  |
| Nearly every day | 2 (2.8%) | 2 (1.4%) | 3 (2.1%) |  |  |  |
| **Thoughts that you would be better off dead or of hurting yourself in some way** |  |  |  |  |  |  |
| Not at all | 65 (90.3%) | 130 (90.3%) | 130 (90.3%) |  | 0.559 | 0.877 |
| Several days | 3 (4.2%) | 9 (6.3%) | 8 (5.6%) |  |  |  |
| More than half the days | 2 (2.8%) | 4 (2.8%) | 4 (2.8%) |  |  |  |
| Nearly every day | 2 (2.8%) | 1 (0.7%) | 2 (1.4%) |  |  |  |

^1^PHQ-9: Patient Health Questionnaire

^2^Chi-Square test
